# A new theoretical performance landscape for suction feeding reveals adaptive kinematics in a natural population of reef damselfish

**DOI:** 10.1242/jeb.243273

**Published:** 2022-07-04

**Authors:** Roi Holzman, Tal Keren, Moshe Kiflawi, Christopher H. Martin, Victor China, Ofri Mann, Karin H. Olsson

**Affiliations:** 1 School of Zoology, Faculty of Life Sciences, Tel Aviv University, Tel Aviv 69978, Israel; 2 Department of Life Sciences, Ben Gurion University, Beer Sheva 8410501, Israel; 3 The Inter-University Institute for Marine Sciences, PO Box 469, Eilat 88103, Israel; 4 Department of Integrative Biology, and the Museum of Vertebrate Zoology, University of California, Berkeley, CA 94720, USA

**Keywords:** Planktivory, Adaptive landscape, Performance space, Functional morphology, Biomechanics

## Abstract

Understanding how organismal traits determine performance and, ultimately, fitness is a fundamental goal of evolutionary eco-morphology. However, multiple traits can interact in non-linear and context-dependent ways to affect performance, hindering efforts to place natural populations with respect to performance peaks or valleys. Here, we used an established mechanistic model of suction-feeding performance (SIFF) derived from hydrodynamic principles to estimate a theoretical performance landscape for zooplankton prey capture. This performance space can be used to predict prey capture performance for any combination of six morphological and kinematic trait values. We then mapped *in situ* high-speed video observations of suction feeding in a natural population of a coral reef zooplanktivore, *Chromis viridis*, onto the performance space to estimate the population's location with respect to the topography of the performance landscape. Although the kinematics of the natural population closely matched regions of high performance in the landscape, the population was not located on a performance peak. Individuals were furthest from performance peaks on the peak gape, ram speed and mouth opening speed trait axes. Moreover, we found that the trait combinations in the observed population were associated with higher performance than expected by chance, suggesting that these combinations are under selection. Our results provide a framework for assessing whether natural populations occupy performance optima.

## Abstract (precis)

**Highlighted Article:** The *in situ* feeding performance of *Chromis viridis* indicates that the population resides close to a local performance peak.

## INTRODUCTION

Understanding the relationships between form and function is a primary goal in evolutionary biology. However, understanding the relationships between morphology, physiology and performance when multiple traits need to be integrated to successfully complete performance tasks is daunting. For example, in order to feed on evasive zooplankton, a predatory fish needs to swim towards it without provoking the prey's escape response and, at the right distance, rapidly open its mouth and expand the buccal cavity to generate suction flows that draw the prey into the mouth. In this context, the performance of the predator can be defined as the ability to successfully capture prey, measured with respect to a gradient in the prey's escape capacity. Successful capture is determined by the complex and nonlinear interaction of numerous functional traits that determine performance, limiting the usefulness of linear multivariate approaches for understanding form-function relationships. Biomechanics provide a mechanistic framework to understand how musculoskeletal design affects body movements and generate hypotheses regarding the effects of different phenotypes (i.e. fin length or mouth size) on the organism's performance. A biomechanic framework is useful for evolutionary biology because it can predict how traits should respond to selection on performance (Arnold, 1983, 2003; Lande and Arnold, 1983), and has guided the investigation of traits that underlie adaptive radiation and diversification (e.g. Warheit et al., 1999; Calsbeek and Irschick, 2007; Mahler et al., 2013; Hulsey et al., 2019).

One approach for investigating the performance consequences of phenotypic variation consists of measuring the performance of multiple individuals and then applying a statistical model that summarizes the effects of different trait phenotypes and their interaction on performance (Arnold and Bennett, 1988; Jayne and Bennett, 1990; Ghalambor et al., 2003). This model can be used to predict the performance at each point in trait space, hereafter defined as the ‘performance landscape’ (Arnold, 2003). However, this experimental approach has several constraints (*sensu* Arnold, 2003). First, inference from the landscape is limited to the range of phenotypes that exist in the study population, and their combinations, not permitting extrapolation to other species. Second, estimation of the landscape's features is prone to larger errors at the edges of the performance space or in regions where fewer observations exist. Third, extreme values are also likely to bias the estimation. And fourth, statistical inferences could be biased by the choice of function fitted to the data.

An alternative approach stems from progress in biomechanical theory and computational methods that are used to mechanistically model many aspects of performance such as swimming (Sfakiotakis et al., 1999; Fish and Lauder, 2006; Liao, 2007), running (Kingma et al., 1996; Minetti, 1998; Barasuol et al., 2013) and armor strength (Polly et al., 2016; Stayton, 2019), among other examples. These models are based on first principles of biomechanics and dynamics, and produce reliable estimates of performance given a set of phenotypic (often kinematics and morphological) trait values that are used as input variables. Because such mechanistic models can mathematically solve for performance given any input values, it is possible to predict theoretical performance outside the trait ranges and combinations observed within the population. Furthermore, the data used to generate the landscape are independent of the population, enabling one to generate hypotheses regarding the location of the population relative to the landscape. This approach has been particularly useful in understanding trait distribution within clades (Tseng, 2013; Dickson and Pierce, 2019; Stayton, 2019; Rader et al., 2020; Polly, 2021; Dickson et al., 2021), providing insights regarding the evolutionary forces that drive the mapping of species on the landscape. However, whether the phenotypes within populations map to a performance peak or whether performance can be improved by selecting specific trait combinations (Anderson et al., 2020) is much less clear.

Here, we take advantage of an existing theoretical framework for inference of suction-feeding performance (Olsson et al., 2020) to investigate the mapping of *in situ* feeding kinematics onto a performance space in a natural population of reef zooplanktivores. This model incorporates six key kinematic measurements from high-speed videos of suction-feeding strikes in fishes. The mechanism of suction feeding consists of a swift opening of the mouth and expansion of the oral cavity, which generates a flow of water into the predator's mouth. Prey that cannot withstand the force of the flow are sucked into the fish's mouth with the surrounding water (Holzman et al., 2012; Day et al., 2015). Observations, experiments, and hydrodynamic modeling reveal that feeding performance is determined by multiple morphological and biomechanical traits. These traits include the size of the mouth, the speed of mouth opening, and the speed of closing the distance between the predator and prey through forward swimming and jaw protrusion (Holzman et al., 2008b, 2012; Day et al., 2015; Olsson et al., 2020). Many zooplankton species are able to evade these predation attempts by sensing the hydrodynamic disturbance caused by the approaching predator and the suction flows (Fields and Yen, 1997; Buskey et al., 2002; Heuch et al., 2007). Thus, strong feeding performance, from the predator's standpoint, is the ability to capture zooplankton by minimizing hydrodynamic disturbance (Viitasalo et al., 1998; Holzman and Wainwright, 2009; Gemmell et al., 2014). This predator–prey dynamics during suction feeding can be treated as a hydrodynamic interaction between a solid particle (the prey) and unsteady flows (the suction flow), and can be modeled by tracking the forces exerted on the prey and its trajectory during the strike (Holzman et al., 2012; Day et al., 2015; Olsson et al., 2020). This hydrodynamic modeling allows us to quantitatively estimate the performance consequences of any given trait combination and reconstruct a performance landscape independent of the observed population for any phenotype (i.e. any combination of values for the input traits).

Our goal was to place kinematic trait distributions in a natural population into our theoretical suction-feeding performance space and determine whether the population was located on a peak, ridge or valley, or on a slope connecting these features. We first constructed and visualized the performance landscape using a mechanistic model of suction feeding (Holzman et al., 2012; Olsson et al., 2020). We then used *in situ* high-speed 3D observations of fishes from a wild population of *Chromis viridis*, a coral reef damselfish that feeds predominantly on zooplankton using suction feeding. We estimated the values of six key kinematic traits for 110 individuals as they fed, undisturbed, on zooplankton in the natural reef environment. We then mapped the population's location with respect to the topographic features of the six-dimensional performance landscape. Specifically, we: (1) explored the landscape's topography to identify local performance peaks; (2) tested whether the performance of the population corresponded to a random distribution of individuals with respect to local performance peaks; (3) determined the trait axes on which individuals deviate most strongly from the nearest peak; and (4) identified the role of trait correlations in determining or constraining performance.

## MATERIALS AND METHODS

### Study system

*Chromis viridis* Cuvier 1830 is a species of damselfish (Actinopteri: Pomacentridae), a family consisting of ∼385 species from 29 genera. Damselfish are chiefly marine, and are generally associated with coral reefs and their adjacent habitats. *Chromis viridis* is a common Indo-pacific coral reef species, found at depths of up to 30 m, with adult standard length ranging from 27 to 59 mm (Allen and Randall, 1980). This species is an exclusive zooplanktivore, using suction feeding to capture drifting prey over the coral reefs (Coughlin and Strickler, 1990; Carassou et al., 2008). Individuals are usually found inhabiting branching corals in schools of a few dozen to a few hundred individuals, feeding while swimming in the vicinity of their home coral during the day and sleeping among its branches at night (Goldshmid et al., 2004).

### Data acquisition

**Fig. 1.**
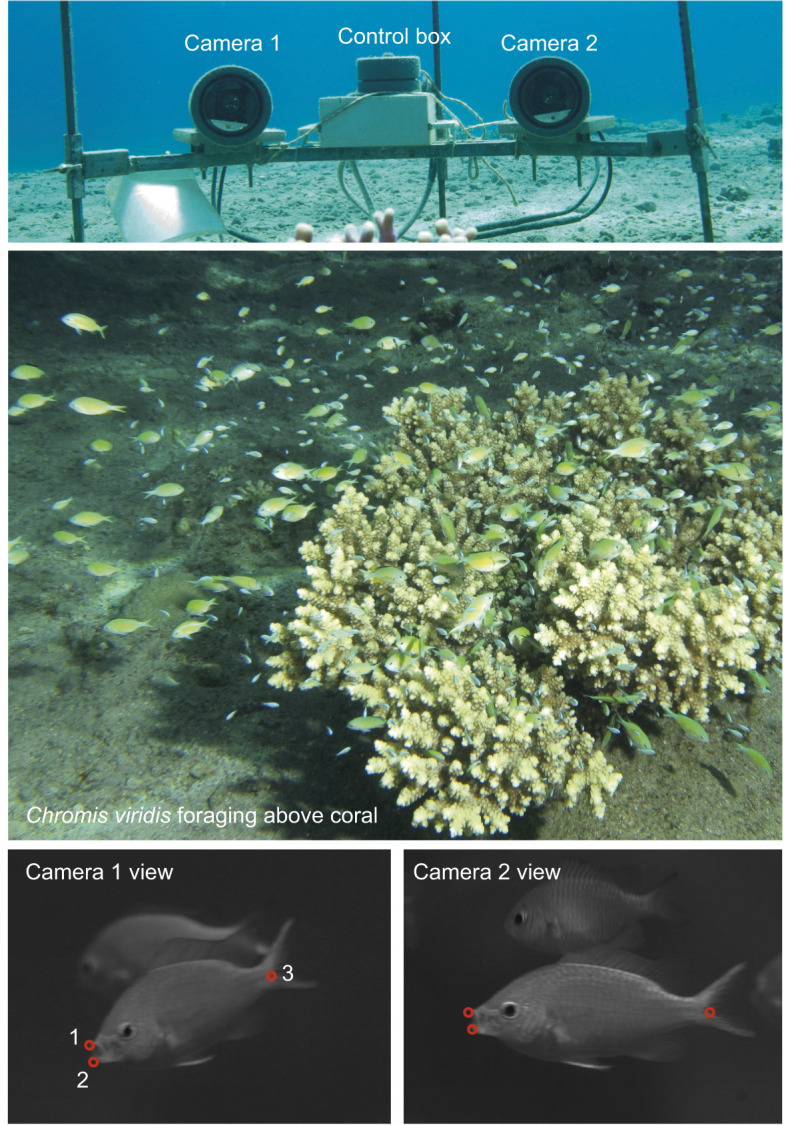
**Underwater filming system.** The filming system at the coral reef in Eilat (upper panel) was set up to film the zooplanktivorous *Chromis viridis* during natural foraging (middle panel). The system captured stereoscopic views of the feeding fish, observed with the two cameras (lower panels). Landmarks (red circles 1–3 in lower panels) were digitized on the fish's body and head to estimate strike kinematics at 500 frames s^−1^.

We filmed prey-acquisition strikes (hereafter ‘strikes’) of *C. viridis* feeding on naturally occurring drifting zooplankton in their natural habitat. All video sequences were recorded over a period of 3 months, from November 2013 to January 2014. Videos were recorded during daytime, using natural light, as these fish are visual predators and are only active during the day. Filming was conducted using synchronized high-speed cameras in a waterproof housing (500 frames s^−1^ at 1.3 megapixels; Hispec1, Fastec Imaging, San Diego, CA, USA). The underwater video system (Perevolotsky et al., 2020; Fig. 1) was moved between five schools, each consisting of tens to hundreds of individuals. All schools were within an area of ∼200×40 m, on the fringing coral reef in front of the Inter-University Institute in Eilat, Israel. The cameras were positioned at an angle of ∼30 deg with respect to one another, and were located ∼1 m from the coral inhabited by the focal fish school. The two cameras were aimed at the same point in space, ensuring multiple views on objects in the visualized volume. Cameras were calibrated using DLTdv5 (Hedrick, 2008), providing a three-dimensional reconstruction of feeding strikes and enabling time-resolved measurements of distances and locations in a volume of ∼20×25×10 cm. The system was re-calibrated every filming session, and measurement error (<1%) was estimated by filming a ruler that was moved within the visualized volume. Live video feed from the camera was viewed onshore through a tethered cable. A trained observer manually triggered the system to record voluntary prey-acquisition strikes of the naturally foraging fish each time such a strike was observed.

**Fig. 2.**
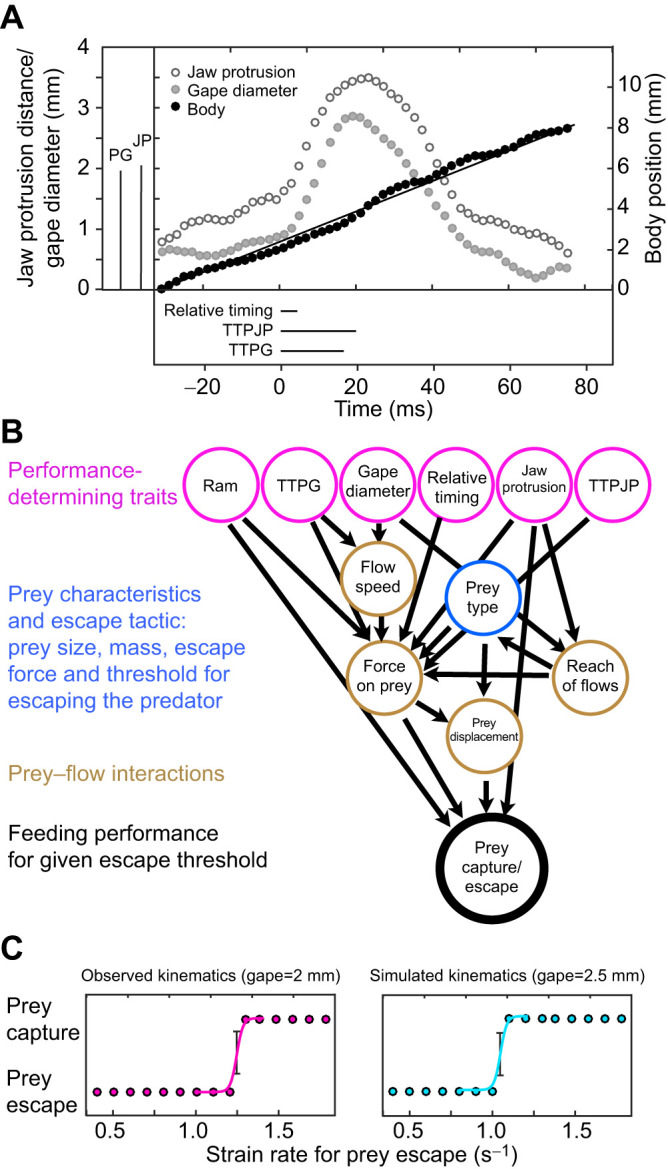
**Using SIFF to estimate prey capture performance from field-observed and simulated kinematics.** (A) With an underwater high-speed 3D camera system (Fig. 1), we recorded body and mouth kinematics of *Chromis viridis*. (B) From these, we calculated a set of six functionally relevant trait values (pink circles; values for the presented strike are indicated in boxes to the left and bottom of the main panel in A). These trait values, in addition to the prey's trait values (blue circle), were used as input variables for SIFF, a hydrodynamic model of prey capture in fish. The model calculates the displacement of the prey in space and time, based on the forces exerted on it by the suction flows (generated by the fish) and the prey's propulsive force. For each set of input parameters, the outcome (prey escape/capture) is deterministic. (C) To estimate performance, the model was run iteratively for each set of input parameters while increasing the strain rate threshold for prey escape. At a sufficiently high threshold (causing a delay in prey escape), the prey is captured, and that threshold is recorded. This process can be carried with observed (pink points) or simulated kinematics, for example using a larger gape diameter (navy circles). The strain rate threshold (±CI) was estimated by fitting a logit function through this data (pink and turquoise curves); in this example, yielding a threshold of 1.275 and 1.08 s^−1^ for the observed and simulated kinematics, respectively. To reconstruct the performance landscape, we used 3291 trait sets, generated computationally by sampling at random from a range of observed trait values in the field population.

For each recorded strike (defined as the sequence of events from the onset of mouth opening to mouth closing), we digitized three landmarks on the body of the fish: the tip of the upper jaw, the tip of the lower jaw and the base of the caudal fin (Figs 1 and 2A). From these landmarks, we calculated the size of the mouth, the location of the mouth center and the location of the fish's body at each frame. From these measurements, we derived the values of six kinematic traits that are known to be important in determining suction feeding performance: (1) maximum gape, defined as the maximum diameter of the fish's mouth; (2) time to peak gape (TTPG), defined as the time to open its mouth from 20% to 95% of peak gape; (3) maximum jaw protrusion measured during the strike from the fish's frame of reference; (4) time to peak jaw protrusion (TTPJP), defined as the time it took the fish to protrude its jaws from 20% to 95% of maximum jaw protrusion; (5) ram speed (*U*_ram_), defined as the fish's swimming speed during the strike (i.e. from mouth opening to closing); and (6) timing of peak protrusion, defined as the difference between the time of maximum gape and time of maximal jaw protrusion (Fig. 2A). Calculations were made as described in Holzman et al. (2008a). Overall, we analyzed a total of 110 strikes in which we could clearly identify and digitize the landmarks.

The high ratio (∼20:1) between the number of fish and the number of sequences recorded in each school translated to a low probability of repeatedly filming the same individual. To further verify data independence, we digitized for each fish the base of the caudal, dorsal and pelvic fins and used these data to estimate the length and body depth of each individual. Using the measurement error obtained by repeatedly measuring the same fish in several points in space, we calculated the confidence intervals for the measurements of the length and body depth (±2 mm). We then assessed the overlap in size (length and body depth) between all the fish observed in our recordings. We assumed that when an individual is recorded twice, the measures of its length and body depth in the one movie would be within the confidence intervals of these measurements in the other movie. This analysis is conservative because there could be multiple individuals with the same length and body depth. Overall, less than 10% of the strikes in each school were performed by individuals of the same size. Therefore, we regarded the strikes as true replicates.

### Computational approach

The procedure of estimating feeding performance based on strike kinematics and generating a performance landscape is described in detail in Olsson et al. (2020) and will be explained here in brief. The first stage was generating a set of 3291 simulated strikes. For each simulated strike, each trait value was sampled at random from a uniform distribution spanning the range of the trait observed in the population. Then, the performance of each strike was assessed using a predictive model (SIFF; see below). This procedure generated performance estimates in 3291 random locations in the six-dimensional trait space. Then, we fit a continuous function to the data using a generalized additive model (GAM) from the mgcv package in R (Wood, 2011), with univariate smoothing splines for each of the six kinematic traits, enabling the prediction of performance in any location at the six-dimensional kinematic trait space. Finally, we verified that the GAM can be a good predictor of the data (Smith et al., 2021). These stages were carried out as follows.

#### Using the suction-induced flow field (SIFF) model to estimate feeding performance

Performance for each of the of the 110 observed strikes as well as the 3291 simulated ‘individuals’ was evaluated using the suction-induced flow field (SIFF) model (Holzman et al., 2012). SIFF uses a set of parameters (Fig. 2B) that characterize the prey and describe the kinematics of the mouth and flow speed during the strike to predict the motion of the prey relative to the mouth during suction feeding (Holzman et al., 2007, 2012; Wainwright and Day, 2007). The value for each kinematic trait in the simulated strikes (max gape, time to peak gape, maximal jaw protrusion, time to peak jaw protrusion, ram speed and timing of peak protrusion) was determined by sampling, at random, from the range of values of the phenotypic traits observed in the population. As such, the location of the simulated strikes (Fig. S1) was unconstrained by the distribution or interdependence between trait values manifested in the observed strikes (Smith et al., 2021).

SIFF is described in detail in Holzman et al. (2007, 2012) and Wainwright and Day (2007). In brief, SIFF uses the flow field realized during the strike and a set of parameters that characterize the prey, the body and the mouth to predict the motion of prey relative to the mouth during suction feeding, given the suction flow generated by the fish and the ability of the prey to move away from it (Fig. 2B). According to SIFF, the movement of the prey relative to the predator's mouth determines whether prey is captured. The total force exerted on the prey is the sum of five component forces: drag, acceleration reaction force, the force resulting from the pressure gradient across the prey, prey swimming forces and gravitational forces. These forces result from the differential in speeds and accelerations between the prey and the water around it, as well as from the gradient of flow across the prey (Holzman et al., 2007, 2012; Wainwright and Day, 2007). The SIFF model was verified against laboratory measurements of suction forces of bluegill and largemouth bass (Holzman et al., 2007, 2008c) and the ability of SIFF to predict dietary abilities of different fishes was verified by comparing the model's predictions with stomach content data for 18 centrarchid species (Holzman et al., 2012).

Flow visualization studies (Holzman et al., 2008a; Day et al., 2015; Jacobs and Holzman, 2018) revealed that the flow field in front of the mouth can be estimated based on mouth kinematics. We used the linear speed of mouth opening *S*_G_ (m s^−1^; the derivative of gape with respect to time during mouth opening) to estimate peak flow speed at the center of the mouth *S*_F_ (m s^−1^) as follows:
(1)

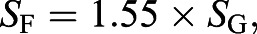
based on the relationships observed in Holzman et al. (2008a) and Jacobs and Holzman (2018). The temporal pattern of the flow was assumed to follow the gape cycle, with flow starting at 20% and peaking at 95% of maximal gape (Holzman et al., 2008a; Day et al., 2015; Jacobs and Holzman, 2018). Based on these flow visualization studies, flow speed at any given point in space can be estimated given the flow speed at the mouth center and the instantaneous gape diameter (Holzman et al., 2008a; Day et al., 2015; Jacobs and Holzman, 2018). From this data, we derived the temporal and spatial flow gradients, and calculated the force exerted on the prey. Prey capture performance was defined based on the sensitivity level of the simulated prey that enabled its escape. We note that prey sensitivity is quantified using strain rate (s^−1^) such that strikes that are associated with higher performance are those that resulted in capture of more sensitive prey (lower strain rate threshold; Fig. 2B,C) (Holzman et al., 2007, 2012; Wainwright and Day, 2007).

A widespread predatory strategy across teleost fishes to rapidly reduce the distance to the prey is to advance the mouth towards the prey, realized by swimming (*U*_ram_) and jaw protrusion (Longo et al., 2016). In the context of feeding on evasive zooplankton prey, swimming incurs a cost, because the advancing body produces a hydrodynamic disturbance (sometimes referred to as the ‘bow wave’). Similar to the suction flows, the bow wave can be sensed by the prey to trigger an escape response (Yen et al., 1992; Fields and Yen, 1997; Stewart et al., 2013). Hydrodynamic disturbance was quantified as strain rate, which can be understood as the rate of deformation of the flow with respect to time. Strain rate is a useful metric for hydrodynamic disturbance because higher strain rates cause faster deflection of sensory antennae of copepods (and sensory systems of other marine invertebrates), ultimately triggering their escape response (Yen et al., 1992; Fields and Yen, 1997). We used observed movements of the body and mouth center as SIFF inputs for the distance between the prey and predator and the properties of the bow wave. The relationship between swimming speed and the hydrodynamic disturbance generated by the body was estimated by visualizing the flow field in front of a formalin-fixed *C. viridis* specimen (standard length=50 mm) held at different flow speeds (2.5–20 cm s^−1^; *n*=10 speeds) in a recirculating flume. Flow visualization and analysis followed the protocol described in Holzman and Wainwright (2009), except that here the fish was mounted in the flume and not free swimming. These measurements showed that strain rate increased with increasing swimming speed and decreased with the distance to the prey, following the equation:
(2)

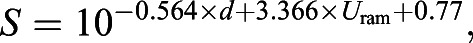
where *S* is the strain rate (s^−1^) at a distance *d* (mm) from the mouth, and *U*_ram_ is the swimming speed (m s^−1^).

Prey was modeled as a naturally buoyant prolate spheroid (0.1 mm in peak diameter, 2 mm length) located on the centerline across from the mouth. The initial location of the prey at the onset of simulation was determined as the point in space where the mouth center would be at the time of peak gape. The prey was modeled to escape directly away from the predator. Peak escape force (2×10^−5^ N), escape duration (10 ms) and latency (1 ms) were adopted from Buskey et al. (2002) and Buskey and Hartline (2003). The prey was modeled to escape once it sensed a hydrodynamic disturbance that exceeded a given strain rate threshold. Strain at the location of the prey was calculated as the sum of strain rates resulting from the advancing body and the suction flows (i.e. we did not account for a possible interaction between the two sources of flow).

For each set of parameters, SIFF determines whether the prey was captured or escaped. To estimate feeding performance, we iteratively ran SIFF for each set of parameters starting with an unrealistically sensitive prey, which escaped when strain rate exceeded 10^−2^ s^−1^_._ This threshold ensured that all prey would escape in the first iteration. We then increased the strain rate threshold by 5% (thus decreasing prey sensitivity) and re-ran SIFF until the prey was captured (Fig. 2C). We fitted a logit function to find the inflection point and its corresponding binomial confidence interval. This value was used as an estimate of the most sensitive prey that could be captured for a given set of kinematic trait values, our metric of zooplankton feeding performance of a zooplanktivorous fish.

#### Estimating the contours of the performance landscape

Phenotypic data and performance estimates of the simulated population were used to estimate a continuous performance landscape, describing the relationship between phenotypic trait values and feeding performance in the complex, multivariate mechanism of suction feeding (Olsson et al., 2020). To estimate the topography of the performance landscape, we used a multivariable GAM with penalized cubic regression splines, and shrinkage smoothing to further penalize highly smoothed terms (Wood et al., 2015). Shrinkage smoothing reduces the estimated effect of parameters with little explanatory power. Cubic regression splines were chosen over thin plate regression and simple multiple linear regressions as they provided the best fit in terms of deviance explained and prediction of SIFF results. We used GAM to fit splines that represent, in the multivariate space, the linkage between the values of six kinematic traits across 3291 simulated individuals with their feeding performance. Given a model structure specified by a GAM formula, the GAM function attempts to find the appropriate smoothness for each spline term (Wood et al., 2015). The fitted model had an adjusted *R*^2^ of 0.899, and explained 90.4% of the deviance. Of the six traits and their paired interactions, only two interactions (maximum jaw protrusion with both TTPG and gape–protrusion time difference) were non-significant and of small effect size.

To verify that the GAM surface provides a reliable approximation of the SIFF results, we divided the dataset into training/test datasets, containing randomly sampled 3181 trait sets used to reconstruct the landscape (training set) and 110 randomly sampled trait sets used to test it (test set). We then calculated, for the test set only, a linear regression of strain rate thresholds derived from SIFF versus those derived from the landscape. The process was repeated 100 times. The mean (±95% confidence interval, CI) intercept and slope of that regression were −0.004±0.33 and 1.009±0.015, respectively, and the *R*^2^ was 0.88±0.05. Given that the 110 trait combinations of the test set were not used to compile the performance landscape, we concluded that this model is suitable to predict feeding performance for any given phenotypic combination within the observed phenotypic ranges.

Finally, we assessed data saturation (Faulkner and Trotter, 2017; Olsson et al., 2020; Smith et al., 2021) by sub-sampling the dataset to obtain subsets of smaller sample size. For each subset, we fitted the same model and carried out a linear regression of fitted against observed values. We then plotted mean-squared error (MSE) of the linear regression against sample size. When sampling across the performance volume is sufficient to capture most of the ruggedness of the landscape, the variance of the MSE should decrease as MSE converges on the error variance. We found that for our models, a dataset with ∼3000 points produced a saturated model.

#### The location of performance peaks on the landscape

To identify the location of the local performance peak(s), we used the gradient ascent method described in Olsson et al. (2020). Briefly, this algorithm ‘hikes’ the landscape towards the path of steepest performance ascent (i.e. decreasing strain rates) until reaching a local peak. To account for the error associated with the SIFF estimates, we generated 50 GAM surfaces, by sampling from the distribution defined by the logit estimate and its confidence interval, and ran all subsequent analysis on these 50 GAM surfaces. The algorithm started in 250 random points across the landscape (five in each landscape), which were deemed a sufficient number by Olsson et al. (2020), and identified several putative local peaks. The algorithm then uses cluster analysis to group nearby local peaks (Olsson et al., 2020). We used the NbClust command in NbClust package using index=all to determine the relevant number of clusters based on several indices of cluster performance. The gradient ascent method (Olsson et al., 2020) identified three peaks, one of them consistent with the global peak. That peak was also independently identified as the stationary point of the response surface, using response surface modeling in the RSM package (Lenth, 2009).

### Question 1: is the population located on a performance peak?

We quantified the distance of the population from the peaks in two complementary approaches: (1) performance-wise, we compared the distribution of strain rate thresholds in the observed population with strain rates estimated for at the local and global peaks, and (2) in trait space, we quantified the average distance of each individual to the nearest peak. We then asked which trait(s) contribute the most to that distance, and what the expected increase in performance is if the individual moves towards the peak on each trait axis. We reasoned that if all individuals are on the peak, the distribution of strain rate values should encompass the value reported for the peak, the average distance to the peak in trait space would be zero, and the average distance to the peak would be shorter than the average distance between individuals (Cheng, 1995).

Because the trait values have different units for different traits, we first standardized and normalized each trait in the observed population by subtracting the mean and dividing by the trait's s.d. We repeated the procedure for the peak location, using the observed mean and s.d. The distance of each individual to the performance peak was calculated as the square root of the sum of square distances for the six (standardized) trait values. Calculations were repeated for the 50 landscapes. In addition, to account for the error in the GAM estimates, we re-evaluated each point estimate by sampling from the distribution defined by the point estimate and its standard error from the predict.gam command. This procedure was repeated 20 times for each point on each landscape (overall 1000 replicates), providing non-parametric confidence intervals to each parameter.

### Question 2: is performance different from that expected at random?

The observed population might be off the peak, yet it still may be located in an area of high performance. To test whether the feeding performance of the observed population is significantly higher than that expected by chance, we generated a ‘null expectation’ for performance by simulating a population of 110 individuals that were generated by drawing, at random, trait values from a normal distribution with a mean and standard deviation equal to the observed population's mean and standard deviation. Values for each trait were selected independently of values selected for the other traits, resulting in an uncorrelated data structure. We repeated this 1000 times (50 on each landscape and 20 for each point as explained above), and calculated the median feeding performance for each simulated population by projecting their trait values on the performance landscape. If the mapping of individuals in the observed population is random with respect to the features of the landscape, we would expect that the median suction performance in the observed population would fall within the 95% CI of the distribution of the median performances of the simulated populations, whereas if the realized phenotypes in the observed population show an increased performance, the median suction performance of the population should exceed the 95% CI of the simulated populations.

### Question 3: do trait correlations constrain or augment performance?

Trait correlations are often suspected to constrain performance. For example, biomechanical coupling of mouth opening and jaw protrusion speeds could limit the realized distribution of these performance-determining trait values to off-peak locations. We tested the role of trait correlations in determining or constraining performance using two complementary approaches.

First, we generated 1000 populations whose individuals were simulated by sampling from a multivariate normal distribution, with a correlation structure identical to the observed population. We hypothesized that if the correlation structure between the different trait values enhances suction performance, the median suction performance of the observed population should overlap the distribution of the simulated population, and exceed the distribution of the simulated constraint-free population (uncorrelated, or ‘null expectation’ population; question 2 above). Alternatively, if the correlation structure constrains suction performance, the median suction performance of the observed population should be outside the distribution of the simulated population, and be lower than the distribution of the simulated constraint-free (uncorrelated) simulated populations.

Second, we sequentially removed the correlation between one trait and the other five and tested the effect of this removal on performance. We did this by randomizing the order of values in one trait (e.g. gape diameter) across the observed population, thereby breaking the correlations between that trait and all the other traits. We then compared the distribution of median performance (strain rate threshold) of the new combinations with that in the observed population. This was done sequentially for each trait. Note that the procedure did not change the correlation structure between the other five traits. We hypothesized that if the correlation structure of the different trait values enhances suction performance, the median suction performance of the observed population should exceed the 95% CI of the simulated constraint-free population. Alternatively, if the correlation structure of the different trait values impedes suction performance, the median suction performance of the observed population should be below the 95% CI of the simulated constraint-free population.

All calculations were performed on the 50 GAM surfaces as explained above.

## RESULTS

### Performance and phenotypic attributes of the observed population

Overall, the morphological and kinematic characteristics of suction feeding in the observed population of *C. viridis* could be described as a swift opening of the mouth (median time to peak gape=24 ms), to a peak diameter of 2.6 mm, with a fast jaw protrusion (median time to peak protrusion=23 ms), reaching peak protrusion distance (median=2.1 mm) 2 ms after the time of peak gape. Ram speed was ∼2 body lengths s^−1^ (median=77 mm s^−1^). With the exception of maximum jaw protrusion distance, all phenotypic trait values were skewed and significantly differed from normal (Shapiro–Wilk, *P*<0.05; Table S1).

Several significant correlations were observed between trait values (Spearman’s rho, ρ>0.2; Table 1), including positive correlations between maximum gape and maximum jaw protrusion (*ρ*=0.59, *P*<0.001), and between TTPG and TTPJP (*ρ*=0.73, *P*<0.001), and negative correlations between TTPG and maximum jaw protrusion (*ρ*=–0.4, *P*<0.001), and between TTPJP and maximum jaw protrusion (*ρ*=–0.37, *P*<0.001).

**Table 1.**
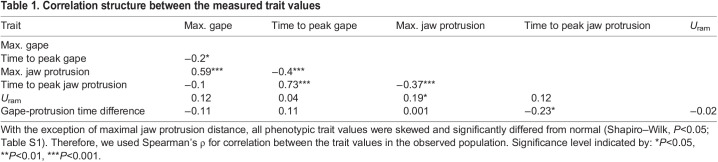
Correlation structure between the measured trait values

### The location of performance peaks on the landscape

**Fig. 3.**
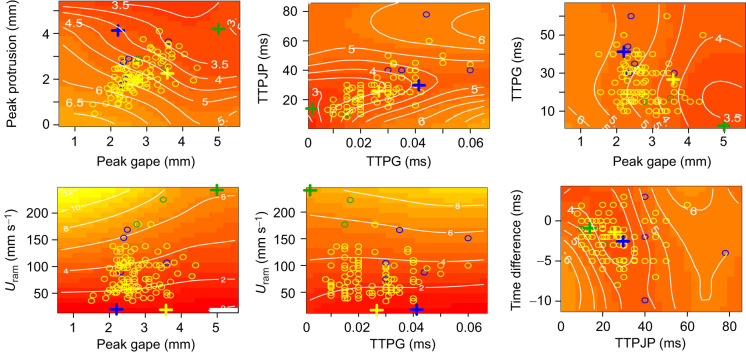
**Two-dimensional projections of the multidimensional performance landscape, generated using a hydrodynamic model of predator–prey interactions.** The multidimensional performance landscape was generated based on 3291 simulated prey-acquisition strikes, featuring random trait combinations selected from the observed trait range. The surface colors (and white contours) represent feeding performance, estimated as strain rate threshold (s^−1^) of the most sensitive prey that can be captured by a predator using that trait combination. Red colors represent high performance (low strain rate thresholds), yellow represents low performance (high strain rate thresholds). Crosses represent the three peaks; blue is the global peak, whereas yellow and green are local peaks. Observed trait combinations (colored circles, *n*=110) are overlaid on the performance surface; circle colors correspond to the closest peak. Note that the 2D visualizations only represent the performance of the featured variables at median values of the other four phenotypic traits, and do not represent trait distribution in the multidimensional space. The plot features six of the 15 possible 2D projections (see Fig. S2 for all projections). TTPG, time to peak gape; TTPJP, time to peak jaw protrusion.

The reconstructed performance landscape revealed a complex, non-monotonic performance space (Fig. 3; Fig. S2) featuring ridges, peaks, valleys and flat surfaces. The density of the contours was different for the different trait combinations, indicating variable performance gradients for each set of trait values. The range of strain rate thresholds estimated across the landscape was ecologically plausible, ranging from 0.1 to 10 s^−1^ (Viitasalo et al., 1998; Kiorboe and Visser, 1999; Visser, 2001; Green et al., 2003; Holzman and Wainwright, 2009), reinforcing the validity of this model in predicting feeding performance based on phenotypic data.

The gradient accent method identified three peaks, one of which was consistent with the location of the global peak from an RSM analysis (Fig. 3, Table 2). The global peak was located at an area of large gape diameter, long protrusion, fast mouth kinematics and slow *U*_ram_, and was outside of the range of parameters of the population (Fig. 3).

**Table 2.**
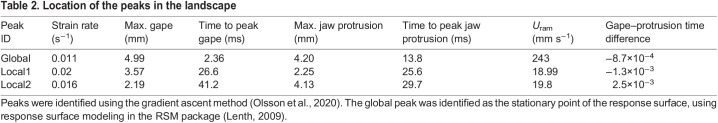
Location of the peaks in the landscape

#### Is the population located on a performance peak?

Most of the individuals in the observed population (*n*=103) were closest in trait space to one of the local peaks than to the other two peaks (Fig. 3). Only five individuals were closest to the global peak, and the remaining two to the other local peak. The scaled distance (in units of s.d.) to the global peak was 7.3±1.2, whereas the distance to the closest local peak was 3.3±0.9. The shortest distance of any individual to the peak in trait space was ∼1.6 s.d. units. The performance associated with all three peaks was much higher than that calculated for any of the observed individuals (i.e. strain rates of <0.02 s^−1^). We therefore reasoned that population is located off the peaks.

#### Is performance different than expected in random?

**Fig. 4.**
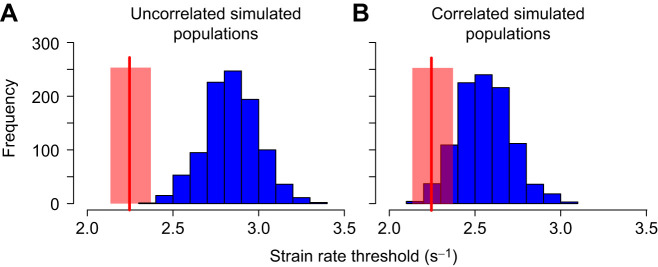
**Median (±95% CI) performance (strain rate threshold) in the observed population (red vertical line±red shaded area), versus the distribution of median performance in two simulated population types.** (A) Populations with no trait correlations; (B) populations with a correlation structure equivalent to that of the observed population. For each scenario (A,B), we simulated 1000 populations, each with 110 individuals, and calculated the median strain rate for each population. The upper CI for the strain rate threshold of the observed population is outside the 95% CI for the uncorrelated null population, but not for the correlated case, indicating that trait combinations play an important part in augmenting suction feeding performance.

Despite being off the performance peak, performance in the observed population was higher than expected by chance. The median strain rate threshold of the observed population (2.24 s^−1^; upper and lower CI=2.12, 2.35) was significantly lower than that expected by chance (mean=2.84, *P*<0.001, 95% CI=2.53–3.15 s^−1^ for the simulated populations; Fig. 4).

**Fig. 5.**
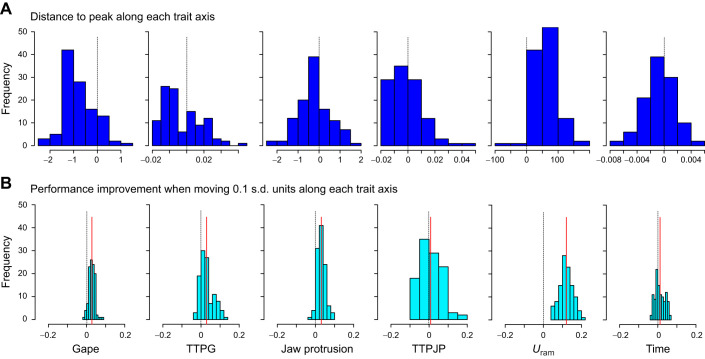
**In general, individuals can deviate from the local peak along any or all trait axes.** We explored this deviation by (A) quantifying the distance to the closest peak on each trait axis and (B) asking how moving towards the peak in each axis contributes to performance. In trait space (A), individuals were furthest on the peak gape (mean±s.d. of 0.8±0.63 mm), *U*_ram_ (63.2±35.1 mm s^−1^) and (to a lesser extent) TTPG axes (2.5±10.9 ms). Movement of 0.1 s.d. units towards the peak (B) had the greatest effect when moving on the *U*_ram_ axis, which improved strain rate threshold by 0.12±0.035 s^−1^ (mean±s.d.), whereas TTPJP and the time difference had the smallest effect on strain rate threshold (0.007 and 0.012, respectively; Fig. 6B). Units in A are the distance to the peak in mm for gape and jaw protrusion, ms for TTPG, TTPJP and time difference, and mm s^−1^ for *U*_ram_. Units in B are improvement in strain rate threshold (s^−1^) for a movement of 0.1 s.d. along each axis. Black vertical lines in A and B mark the 0 point on the *x*-axis; red lines in B are the medians of the population.

In general, individuals can deviate from the local peak along any or all trait axes. We explored this deviation by (1) quantifying the distance to the closest peak on each trait axis and (2) asking how moving towards the peak in each axis contributes to performance. In trait space (Fig. 5A), individuals were furthest on the peak gape (mean±s.d. of 0.8±0.63 mm), *U*_ram_ (63.2±35.1 mm s^−1^) and (to a lesser extent) the TTPG axes (2.5±10.9 ms). Movement of 0.1 s.d. units towards the peak had the greatest effect when moving on the ram axis, improving strain rate threshold by 0.12±0.035 s^−1^ (mean±s.d.), whereas TTPJP and the time difference had the smallest effect on strain rate threshold (0.007 and 0.012, respectively; Fig. 5B).

**Fig. 6.**
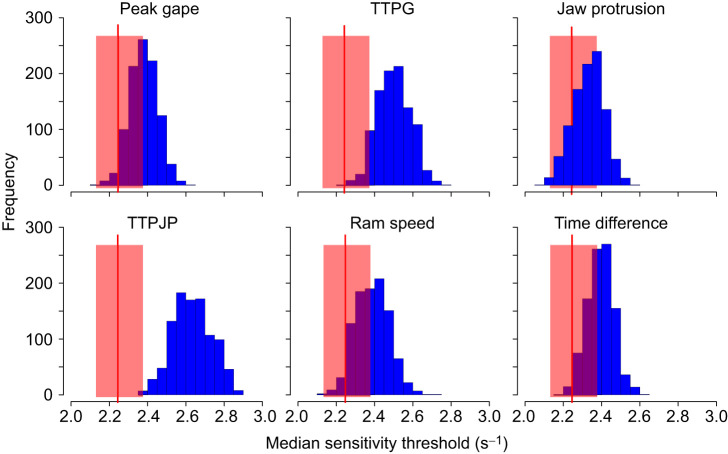
**Breaking trait correlations between one trait and all others reduces performance.** Results depict strain rate threshold in 1000 simulated populations (blue bars) in which we randomized the order of one trait (depicted in each panel), thereby breaking the correlation structure between the focal trait and the other five trait values. The median (±95% CI) strain rate threshold in the observed population (red vertical line±red shaded area) is plotted next to the histogram. Trait values related to jaw kinematics (TTPG and TTPJP) were most sensitive to breaking the correlations with other trait values.

#### Do trait correlations constrain or augment performance?

Of the six traits and their paired interactions, all but two interactions were significant in the GAM model (Table S2), indicating the importance of trait correlations in determining performance. In other words, the GAM analysis reveals that certain trait combination have additive or diminutive effects on performance. To explore this role further, we simulated populations in which trait values had the same correlation structure as the observed population. Trait correlations are often thought of as leading to performance constraints, providing a possible explanation for the off-peak location of the observed population. However, in contrast to this expectation, observed trait correlations contributed to feeding performance. The median strain rate threshold in this simulated population was 2.55 s^−1^ (upper and lower CI=2.27 and 2.89), overlapping the CI of the observed population (Fig. 4B).

We applied a second test for the effect of trait correlation, which consisted of removal of trait correlations for each one of the traits at a time. The motivation for this test was (1) to test the role of correlations while not changing the underlying trait distribution and (2) to understand which trait correlations are more important. The results of this procedure were consistent with the role of trait correlations (combinations) in improving performance, as breaking correlations generally reduced median performance for all six traits (mean strain rates of 2.33–2.65, compared with 2.24 in the observed population). Of the six traits, jaw kinematic traits (TTPG and TTPJP) were the most sensitive to breaking the correlations with other traits (Fig. 6).

## DISCUSSION

We mapped the distribution of performance-determining phenotypic trait values on a landscape derived from a mechanistic model of prey capture performance. Our approach allows a robust interpretation of the performance landscape beyond the ranges observed in the sampled population and curtails ‘edge effects’ that typically result in lower accuracy of the landscape near the population edge. Mapping the observed population on the landscape indicated that a natural population of zooplanktivores was located off the local performance peak, although performance was higher than expected by chance. Nevertheless, our inference indicates that trait correlations did not constrain performance because breaking trait correlations in simulated populations reduced performance compared with the observed population. We conclude that our approach can help uncover the relationships between trait distributions in a population, performance and functional constraints in complex functional systems.

### The population is not located on a performance peak

The performance landscape for suction feeding reported here was complex and rugged, and featured performance troughs and ridges (Fig. 3). The landscape clearly showed that different phenotypic traits have a different effect on performance as indicated by the difference in the contour densities of the performance landscape for each trait (Fig. 3). It is further apparent that the mapping of morphology to performance is complex; for example, for low values of TTPJP (<0.02 s), the effect of TTPG on performance is much stronger than at high values (TTPJP>0.06 s; Fig. 3). These trends highlight the importance of an integrative model to characterize complex functional systems.

Functional systems are often inferred as being ‘optimized’ for performance, i.e. the distribution of trait values within a species or a population is assumed to correspond to performance peak (Gould, 1980; van Leeuwen and Muller, 1984; Arnold and Bennett, 1988; Bishop et al., 2008). This notion is often supported by using the measured performance data to construct a putative landscape (Arnold and Bennett, 1988; Arnegard et al., 2014). More often, and especially under a phylogenetic comparative framework, it is the distribution of trait values that is used to infer the existence and location of a ‘peak’ (Collar et al., 2009; Shoval et al., 2012; Ingram and Mahler, 2013). However, the correspondence (or lack thereof) between the performance landscape and the distribution of species trait values is generally poorly demonstrated, typically because performance is not actually measured. As pointed out by Arnold (2003), it is problematic to infer the location of performance peaks from laboratory or field measurements of performance. Therefore, the location of the population with respect to topography of the performance landscape (e.g. peaks and ridges) is difficult to resolve and is largely unclear. Here, we show that suction-feeding performance of *C. viridis* is not optimal, although it is significantly higher than that expected under a model of random motion across the landscape. We argue that it is unlikely that this sub-optimal distribution is associated with selection imposed on the six traits along an additional performance. This is because *C. viridis* is a zooplankton specialist, feeding mainly on copepods (Allen and Randall, 1980). Unlike other damselfishes, it does not utilize the mouth for tasks such as brooding eggs, nest building or biting/scraping (Smith and Wootton, 1995; Ostlund-Nilsson and Nilsson, 2004), which could impose selection towards other optima. We therefore presume that focusing on a single performance landscape for only strain-sensitive prey is reasonable, and that the functional demands for suction feeding (rather than those for respiration or aggression) impose the strongest direct selection on the trait values we measured.

Instead, we speculate that suction-feeding performance is constrained by mechanical limitations. Our model is purely functional, accounting for the effects of strike kinematics on performance. However, it does not account for energetic, genetic, developmental or biomechanical constraints on the suction feeding mechanism (Van Wassenbergh et al., 2006; Wainwright et al., 2007; Carroll and Wainwright, 2009). Therefore, high-performance trait combinations identified by the model may be unrealistic owing to such constraints. For example, there could be ecological constraints on body size, such as some maximal body size that allows *C. viridis* to inhabit and take shelter in *Acropora* corals, which directly limits muscle mass and thus associated feeding kinematics. Alternatively, biomechanical principles dictate that increases in gape size are likely to increase TTPG (Hulsey and Wainwright, 2002; Oufiero et al., 2012) and increase the energetic cost of the strike (Carroll and Wainwright, 2009). Such couplings are not accounted for by SIFF and are therefore not implicit in the landscape. Similarly, faster TTPJP might permit capturing more sensitive prey (Fig. 3); however, in reality it could be disfavored because it will increase the energetic cost of the strike (Carroll and Wainwright, 2009). Likewise, the relative timing of peak gape and peak jaw protrusion could be a consequence of the biomechanical lever system (Westneat, 2005), having little effect on performance within the observed range of trait values. Genetic linkages are also expected to constrain the distribution of trait values in the observed population (Lande and Arnold, 1983; Schluter, 1996). Nevertheless, in our simulated populations, we accounted for such correlations, which were shown to have a strong effect on the phenotypic distribution of our populations and on performance.

Alternatively, the location of the population off the performance peak could result from a misalignment of the performance and fitness peaks. For example, it could be that the performance peak allows the capture of highly strain-sensitive prey, but the energetic cost of feeding with such morphology is prohibitively high. If the abundance of such highly evasive copepods is very low compared with other (less evasive) copepod species, the fitness peak (or peak energetic gain) will be shifted towards the current location of the population. However, we are unaware of community-wide data on the distribution of hydrodynamic performance of copepods that would enable testing of this idea.

The performance landscape accounted well for the variation in performance that stems from the variation in kinematics. However, it relied on simplifications that do not allow us to account for variation in prey size and taxa, the environment or other individual fish in the school. It could very well be that variation in these factors critically affects the response of the fish we observed. For example, some of the variation in kinematics may be due to changes in prey size and type (Ferry-Graham et al., 2001), which we could not visualize with our system. We also did not account for the effects of water flow and turbulence, which can affect the ability of the predator to locate and reach its prey (Kiflawi and Genin, 1997) and the ability of the prey to detect and respond to the predator (Buskey, 1994; Gilbert and Buskey, 2005). Similarly, inter-school competition may drive individuals to swim faster to get to the prey before other individuals while making greater hydrodynamic noise, possibly sacrificing performance. These, and similar effects, can be incorporated into the landscape, but at the cost of adding more dimensions.

The performance landscape is multidimensional. It is therefore only possible to visualize it in two-dimensional projections (Fig. 3, Fig. S2). Qualitatively examining the distribution of the observed population in these 2D maps can be a useful tool in generating hypotheses regarding the distribution of the trait values on the landscape. For example, the projection showing TTPG and TTPJP (Fig. 3; middle panel) may support the intuition that some trait values are distributed along ‘performance ridges’ (Arnold et al., 2001; Arnold, 2003; Ghalambor et al., 2003; Whibley et al., 2006), along which morphological variation can accumulate without performance consequences. However, it is important to remember that the 2D visualization only represents the behavior of the displayed variables at median (or another pre-defined) values of the other phenotypic traits, and cannot accurately represent trait distributions in the multidimensional space (Gavrilets, 1997; Blonder, 2016).

### Generality of the framework

Following the work of Hansen and Martins (1996), Arnold et al. (2001) suggested that using performance landscapes can be helpful in understanding micro- and macro-evolutionary processes. However, such applications of the performance landscape are rare, specifically at the micro-evolutionary level. At the macro-evolutionary level, several studies calculated a performance surface to map extant and extinct species onto it. For example, Tseng (2013) used finite element analysis to map the values of two traits (skull width-to-length and depth-to-length ratios) to two functional properties, the mechanical advantage and strain energy of the Carnivora skull. That study calculated the strength of theoretical skull shapes to estimate the functional properties of trait values that lay outside the distribution of observed skull shapes, concluding that skull shapes evolved towards higher performance, but also discovering high-performance regions that are not occupied by extant species. Similarly, Polly et al. (2016) used finite element analysis and hydrodynamic theory to predict the trade-off between turtle shell strength and drag, and mapped the distribution of turtle species with respect to the resulting Pareto front. The mapping of species on the performance landscape generally indicates that species are located in areas of high performance, but that the landscape contains other high-performance regions that are not occupied. Our results provide similar evidence for the evolution of feeding morphology on a complex performance landscape as an important process that drives the present intra-specific phenotypic distribution. Field experiments now demonstrate that complex fitness landscapes drive adaptive radiation in rapidly speciating fish groups (Martin and Wainwright, 2013; Arnegard et al., 2014). It will be interesting to compare the topography of these fitness landscapes with the topography of empirical performance landscapes.

Importantly, although our study focuses on suction feeding in fish, the framework we present here is general and widely applicable across taxa and functional systems. Over the last few decades, biomechanical theory and computational methods have been used to mechanistically model many aspects of performance such as swimming (Sfakiotakis et al., 1999; Fish and Lauder, 2006; Liao, 2007), running (Kingma et al., 1996; Minetti, 1998; Barasuol et al., 2013), slithering (Hu et al., 2009), and flying and gliding (Wu, 2011; Paranjape et al., 2012), among numerous other examples. These models can be used to generate performance landscapes and map the distribution of functional traits on these landscapes in a variety of functional systems, and in many species. Because performance is fundamentally connected to viability selection in the wild, this framework now enables us to test how these complex functional systems shape evolutionary trajectories across species.

The performance landscape can be a useful tool to generate predictions regarding the functional consequences of shifting trait means in response to ecological (e.g. predation or competition) and evolutionary (e.g. co-evolution) processes (Arnold et al., 2001; Arnold, 2003; Ghalambor et al., 2003). For example, the performance landscape can predict how removing large individuals with larger gape (e.g. through predation or fishing) will change the distribution of performance in the population. Likewise, performance landscapes can be used to predict the morphological response to evolutionary changes in prey escape capabilities such as increased sensitivity or the ability to accelerate and swim faster. The performance landscape can also be used to better understand the nature of intra-individual variation in strike kinematics. Even when filming fish in the laboratory under controlled conditions, individual fish have considerable variation in kinematics and performance (Holzman et al., 2007; Oufiero et al., 2012; Jacobs and Holzman, 2018), and the landscape can be used to investigate whether this variation is functional (e.g. different kinematics simply suit different prey types) or stochastic (e.g. strikes are scattered around the peak).

Critically, the complex nature of the landscape warrants caution when inferring selection, because (even under the unsupported assumption that the performance landscape aligns with the fitness landscape) selection may operate differently on the same traits in different areas of morphospace, depending on the values of other traits of the system. For example, two individuals that have the same trait value for maximal jaw protrusion can be subjected to different selection regimes, depending on the values of their other traits. The selective force operating on each point in the multi-dimensional space can be calculated as the sum (for all trait values) of the derivatives of performance with respect to changing trait values. It also follows that simple mathematical functions (Arnold et al., 2001) fail to capture the complex nonlinear features of the landscape (Schluter, 1996).

## Supplementary Material

Supplementary information
