# Supplementary material for: A new theoretical performance landscape for suction feeding reveals adaptive kinematics in a natural population of reef damselfish
Source: J Exp Biol. 2022 Jul 4;225(13):jeb243273. doi: 10.1242/jeb.243273 (PMC9339911; doi:10.1242/jeb.243273)
Supplement: Supplementary information [file jexbio-225-243273-s1.pdf]

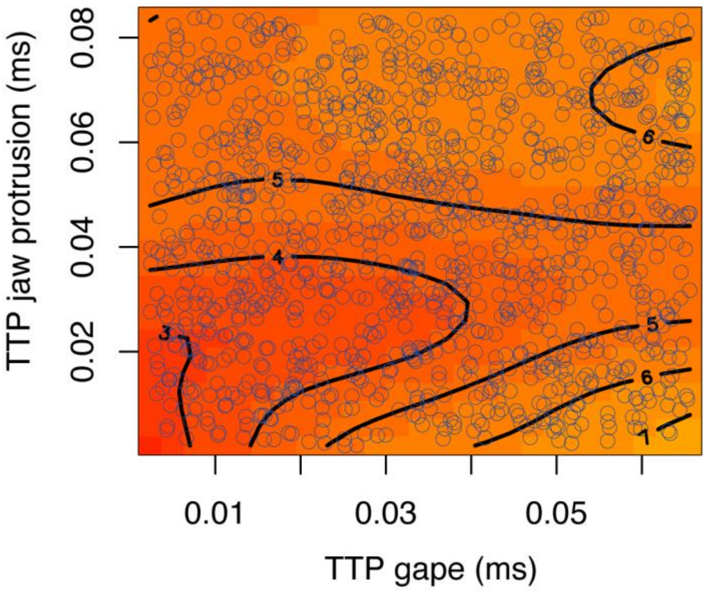

Fig S1. Mapping of the ~3300 phenotypic combinations used as SIFF inputs (open blue circles) to generate the landscape, projected on a 2D representation of the landscape. Note the dense coverage of the landscape. Colors (and black contours) represent feeding performance, estimated as strain rate threshold (s^{-1}) of the most sensitive prey that can be captured by a predator using that trait combination. Red colors represent high performance (low strain rate thresholds), yellow represents low performance (high strain rate thresholds).

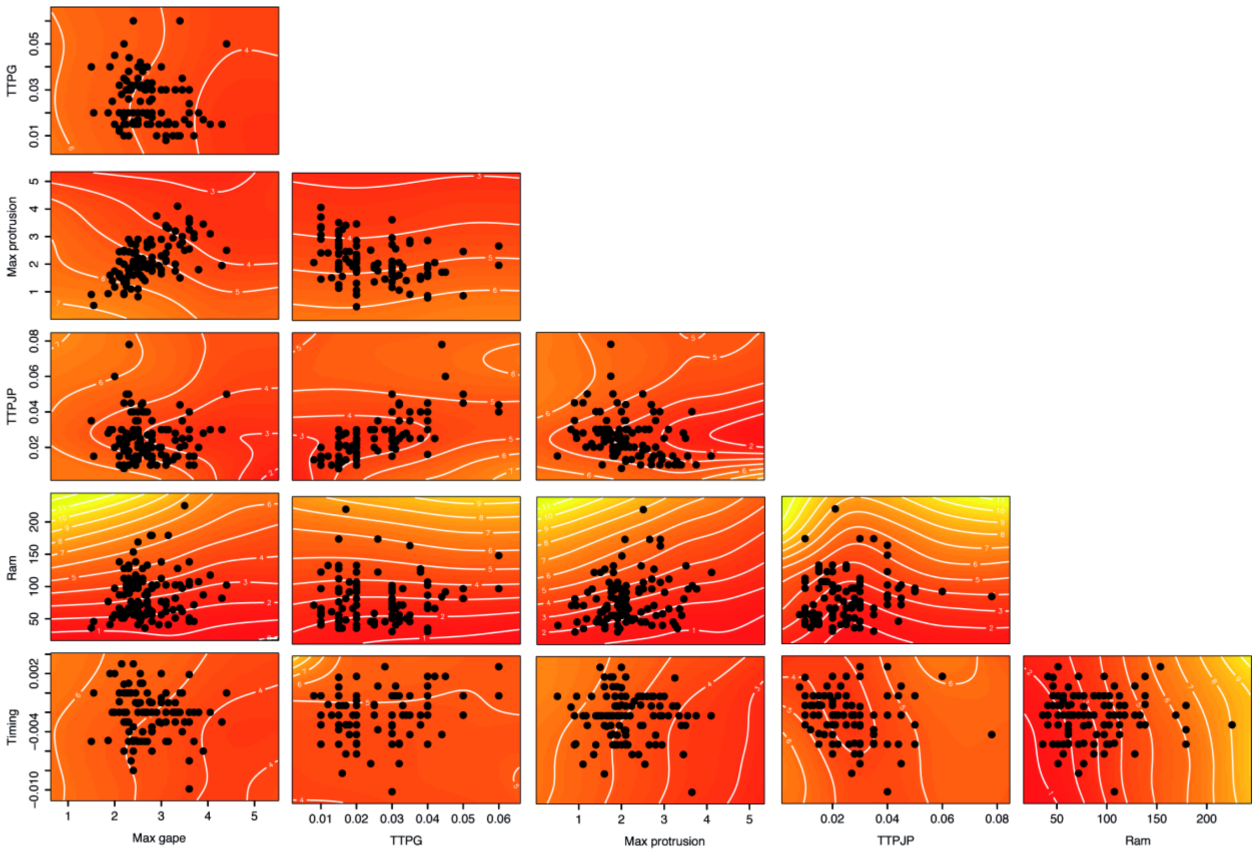

Fig. S2. 2D projections of the multidimensional performance landscape, generated using a hydrodynamic model of predator-prey interactions. The plot features all 15 possible 2D projections. The multidimensional performance landscape was generated based on 3300 simulated prey-acquisition strikes, featuring random trait combinations selected from the observed trait range. Colors (and white contours) represent feeding performance, estimated as strain rate threshold (s^{-1}) of the most sensitive prey that can be captured by a predator using that trait combination. Red colors represent high performance (low strain rate thresholds), yellow represents low performance (high strain rate thresholds). Observed trait combinations (black circles, n=110) are overlaid on the performance surface. Note that the 2D visualizations only represent the performance of the featured variables at median values of the other four phenotypic traits, and do not represent trait distribution in the multidimensional space.

Table S1. Summary statistics for the six morphological traits in the observed population

Trait	Median	Mean	SD	CV	Shapiro-Wilk's test results for normality of distribution
Max gape	2.60 mm	2.73 mm	0.56 mm	0.205	**
Time to peak gape	20 msec	24 msec	11 msec	0.463	***
Max jaw protrusion	2 mm	2.15 mm	0.70 mm	0.328	N/S
Time to peak jaw protrusion	23 msec	25 msec	12 msec	0.485	***
Ram speed	76.8 mm/sec	86 mm/sec	36 mm/sec	0.414	***
Gape-protrusion time difference	-2 msec	-2.1 msec	2.4 msec	1.106	*

Significance level indicated by: * - $p<0.05$, ** -$p<0.01$, *** - $p<0.001$. N/S - $p>0.05$.

Table S2. Contribution of morphological traits and their interactions to the GAM-smoothed performance landscape.

Trait	Effective degrees of freedom	Deviance from null (Wald statistic)	Significance level
Max gape (gape_max)	5.10	211.625	***
Time to peak gape (TTPG)	3.64	60.4	***
Max jaw protrusion (JP_max)	5.82	153.039	***
Time to peak jaw protrusion (TTPJP)	6.72	74.413	***
Ram speed (ram_spd)	5.58	2596.523	***
Gape-protrusion time difference (time_diff_peak)	4.02	7.095	***
gape_max:TTPG	2.48	4.854	**
gape_max:JP_max	7.17	3.981	***
gape_max:TTPJP	10.88	6.721	***
gape_max:ram_spd	10.53	45.606	***
gape_max:time_diff_peak	5.68	2.235	*
TTPG:JP_max	0.00	0.481	N/S
TTPG:TTPJP	11.47	23.131	***
TTPG:ram_spd	7.39	18.293	***
TTPG:time_diff_peak	13.44	12.051	***
JP_max:TTPJP	10.74	12.511	***
JP_max:ram_spd	7.49	33.797	***
JP_max:time_diff_peak	3.27	1.573	N/S
TTPJP:ram_spd	12.22	14.315	***
TTPJP:time_diff_peak	11.58	34.186	***
ram_spd:time_diff_peak	9.20	2.318	**

Significance level indicated by: * - p<0.05, ** -p<0.01, *** - p<0.001. N/S - p>0.05.

Wald statistics and p-values were calculated according to Wood (2013).
